# Therapeutic delivery of siRNA with polymeric carriers to down-regulate STAT5A expression in high-risk B-cell acute lymphoblastic leukemia (B-ALL)

**DOI:** 10.1371/journal.pone.0251719

**Published:** 2021-06-22

**Authors:** Mahsa Mohseni, Cezary Kucharski, Remant Bahadur K. C., Mohammad Nasrullah, Xiaoyan Jiang, Hasan Uludağ, Joseph Brandwein

**Affiliations:** 1 Department of Medicine, Faculty of Medicine and Dentistry, University of Alberta, Edmonton, AB, Canada; 2 Department of Chemical and Materials Engineering, Faculty of Engineering, University of Alberta, Edmonton, AB, Canada; 3 Department of Medical Genetics, Faculty of Medicine, University of British Columbia, Vancouver, BC, Canada; 4 Faculty of Pharmacy and Pharmaceutical Sciences, University of Alberta, Edmonton, AB, Canada; 5 Department of Biomedical Engineering, Faculty of Medicine and Dentistry, University of Alberta, Edmonton, AB, Canada; German Cancer Research Center (DKFZ), GERMANY

## Abstract

Overexpression and persistent activation of STAT5 play an important role in the development and progression of acute lymphoblastic leukemia (ALL), the most common pediatric cancer. Small interfering RNA (siRNA)-mediated downregulation of STAT5 represents a promising therapeutic approach for ALL to overcome the limitations of current treatment modalities such as high relapse rates and poor prognosis. However, to effectively transport siRNA molecules to target cells, development of potent carriers is of utmost importance to surpass hurdles of delivery. In this study, we investigated the use of lipopolymers as non-viral delivery systems derived from low molecular weight polyethylenimines (PEI) substituted with lauric acid (Lau), linoleic acid (LA) and stearic acid (StA) to deliver siRNA molecules to ALL cell lines and primary samples. Among the lipid-substituted polymers explored, Lau- and LA-substituted PEI displayed excellent siRNA delivery to SUP-B15 and RS4;11 cells. STAT5A gene expression was downregulated (36–92%) in SUP-B15 and (32%) in RS4;11 cells using the polymeric delivery systems, which consequently reduced cell growth and inhibited the formation of colonies in ALL cells. With regard to ALL primary cells, siRNA-mediated STAT5A gene silencing was observed in four of eight patient cells using our leading polymeric delivery system, 1.2PEI-Lau8, accompanied by the significant reduction in colony formation in three of eight patients. In both BCR-ABL positive and negative groups, three of five patients demonstrated marked cell growth inhibition in both MTT and trypan blue exclusion assays using 1.2PEI-Lau8/siRNA complexes in comparison with their control siRNA groups. Three patient samples did not show any positive results with our delivery systems. Differential therapeutic responses to siRNA therapy observed in different patients could result from variable genetic profiles and patient-to-patient variability in delivery. This study supports the potential of siRNA therapy and the designed lipopolymers as a delivery system in ALL therapy.

## 1. Introduction

Acute lymphoblastic leukemia (ALL), the most common type of childhood cancer [1], is characterized by overproduction and accumulation of malignant lymphoid progenitor cells within the bone marrow. Current multiagent chemotherapy regimens have improved the treatment outcomes in people under age 20, with 5-year survival rate of 89%. However, outcomes in adults remain inferior, largely owing to the development of chemoresistance leading to relapse [2]. In older adults, severe toxicities limit the ability to administer intensive chemotherapy. In B-ALL, which accounts for 80% of ALL, two molecular subtypes, BCR-ABL^+^ and BCR-ABL-like subgroups, are associated with high relapse rates and inferior survival in both childhood and adult B-ALL [3, 4]. These two subtypes accounts for over 60% of adult ALL in some series [3, 5, 6]. Therefore, developing novel treatment modalities is of utmost importance to improve treatment outcomes in these high-risk patients [7, 8].

Transcription factors are one of the most promising molecular targets for siRNA-based cancer therapy, as multiple oncogenic signalling pathways converge on a limited group of transcription factors which promote cell growth and survival [9]. One such key group of transcription factors is the Signal Transducer and Activator of Transcription (STAT) family proteins. These proteins act as nuclear transcription factors that activate the expression of a diverse set of genes, including some that are involved in cancer cell development, growth and survival [9, 10]. Certain STATs, such as STAT3 and STAT5, are often constitutively active when one or more upstream tyrosine kinases become overactive due to different genetic alterations leading to a variety of solid tumors and blood malignancies [10]. In the normal process of early B-cell development in bone marrow, STAT5 is required to induce cell survival and B-cell expansion [11–13]. Studies in BCR-ABL-like B-ALL cells have demonstrated constitutive activation of a variety of key signal transduction pathways, including JAK, EPOR, ABL and PDGFR. Activation of these pathways induces overexpression of STAT5, leading to uncontrolled proliferation and/or survival of leukemic cells [14]. Similarly, STAT5 is activated indirectly by BCR-ABL1 in Philadelphia (Ph)^+^ B-ALL cells leading to its overexpression [15–17]. In BCR-ABL^+^ chronic myeloid leukemia (CML), high levels of STAT5 mRNA correlate with tyrosine kinase inhibitor (TKI) resistance, regardless of the presence of tyrosine kinase domain (TKD) BCR-ABL1 mutations [18, 19]. A preclinical study in BCR-ABL^+^ and BCR-ABL-like B-ALL cell lines and primary cells derived from newly diagnosed and relapsed/TKI-resistant BCR-ABL-like ALL patients, found that STAT5 silencing suppressed cell growth, induced apoptosis, and inhibited leukemogenesis [3]. These studies suggest that STAT5 signaling is a potentially attractive therapeutic target in high-risk B-ALL [3, 9, 20, 21]; however, targeted small molecule inhibitors of STAT5 are not currently available.

RNA interference (RNAi) has emerged as an alternative approach to inhibit signaling pathways. RNAi is a process by which double-stranded small interfering RNA (siRNA) can induce sequence-specific, post-transcriptional gene silencing [22]. To control the expression of genes involved in these malfunctioning processes, synthetic siRNA can be delivered into diseased cells to interact with the target mRNA of aberrant genes for degradation or inhibition of translation, thereby silencing their expression [22]. However, siRNA therapy requires an efficient delivery system since naked siRNA molecules are susceptible to degradation by endogenous nucleases in serum, and they are not able to pass through cellular membranes due to their anionic nature. Among different delivery systems, cationic polymers are safer carriers than the viral vectors for intracellular delivery of anionic siRNA molecules as they do not have the capacity to integrate into host genome. Moreover, cationic polymers can be chemically engineered and functionalized according to the needs of the application [22–24]. Lipid substituted low molecular weight (MW) polyethylenimine (PEI) is a promising cationic polymer to undertake siRNA delivery into leukemic cells as it effectively condenses siRNA molecules into nanoscale particles due to its high charge density, facilitates cell internalization through electrostatic interaction with plasma membranes, and displays high buffering capacity needed for endosomal escape [25–27]. The lower MW PEI does not display the disadvantages of the high MW (>25 kDa) PEIs such as high cytotoxicity and limited biodegradability. However, low MW PEIs need to be modified for effective siRNA delivery to provide the required stability for the self-assembly process during complexation with polynucleotides and increase the interactions with the plasma membrane to facilitate the cellular uptake. Therefore, we have utilized lipid substitution on the amine groups of low MW PEIs to improve the efficacy of siRNA delivery into the cells [28, 29].

In this study, the impact of STAT5A inhibition on B-ALL cell lines and ALL patient-derived cells were assessed by screening a library of lipid-substituted polymeric nanoparticles and determined the most effective polymeric carriers for STAT5A siRNA delivery. We focused on the silencing activity of siRNA delivery in selected cell models as the physicochemical properties of the lipopolymers were earlier reported [30].

## 2. Materials and methods

### 2.1. Materials

The low MW PEIs (0.6, 1.2, and 2.0 kDa), anhydrous dimethyl sulfoxide (DMSO), formaldehyde, chloroform, 2-Mercaptoethanol, doxorubicin hydrochloride (product number: 44583), and thiazolyl blue tetrazolium bromide (MTT) were purchased from Sigma-Aldrich (St. Louis, MO). Iscove’s Modified Dulbecco’s Medium (IMDM), Roswell Park Memorial Institute Medium (RPMI) 1640 medium with L-glutamine, fetal bovine serum (FBS), GlutaMAX, Hank’s balanced salt solution (HBSS), phosphate buffered saline (PBS), penicillin, streptomycin, and UltraPure DNase/RNase-free dH2O were obtained from ThermoFisher Scientific (Ottawa, Canada). Interleukin 3 (IL3), IL6, IL7, FMS-like tyrosine kinase 3 ligand (Flt3-L), and stem cell factor (SCF) were supplied by PeproTech (Rocky Hill, NJ, USA). BIT 9500 serum substitute was purchased from StemCell Technology (Vancouver, BC, Canada). Negative control scrambled siRNA (Cat. No. DS NC1), the 6-carboxyfluorescein (FAM) labeled scrambled siRNA, STAT5A specific dicer-substrate siRNA (5′-CCCGAUUUCUGAGUCACUAAAGCGCAA-3′ and 3′-GGGCUAAAGACUCAGUGAUUUCGCG5′-5′), and a custom-synthesized BCR-ABL specific dicer-siRNA (5′-GCAGAGUUCAAAAGCCCU-3′ and 3′-GUCUCAAGUUUUCGGGAA-5′) were obtained from Integrated DNA Technologies (IDT) (Coralville, IA, USA). SensiFAST cDNA Synthesis Kit was from FroggaBio Inc. (Toronto, ON, Canada). Luna® Universal One-Step qPCR Kit was ordered from New England Biolabs ® (NEB), Inc. (Ipswich, MA, USA). Human methylcellulose enriched media (Cat. No. HSC005) and human methylcellulose base media (Cat. No. HSC002) were supplied by R&D systems, Inc. (Oakville, ON, Canada). Trizol used for total RNA extraction and Lipofectamine™ RNAiMAX Reagent were from Invitrogen (Carlsbad, CA).

### 2.2. Cell models and cultures

Acute lymphocytic RS4;11 and SUP-B15 leukemia cells were purchased from American Type Culture Collection (ATCC) (Rockville, MD, USA). SUP-B15 Cells were cultured in IMDM supplemented with 20% FBS, 0.05 mM 2-Mercaptoethanol, 100 U mL^−1^ penicillin, and 100 μg mL^−1^ streptomycin. RS4;11 cells were cultured in RPMI 1640 medium supplemented with 10% FBS and 100 U mL^−1^ penicillin, and 100 μg mL^−1^ streptomycin. All cell lines were maintained at 37°C and 5% CO_2_. To sub-culture the cells after reaching 80% confluency, cells were centrifuged at 900 rpm for 5 min and passaged at a 20% concentration of the original count.

Ten frozen ALL patient cells were obtained from the biobank at the University of Alberta Hospital (Edmonton, AB, Canada) with the approval of the institutional Health Research Ethics Board. We obtained written informed consent from subjects for all research samples (there were no minors, only adults). Patient samples with specific genetic abnormalities were selected according to the World Health Organization guidelines for categorizing the ALL subtypes. To culture the mononuclear (MN) cells obtained from ALL frozen samples, the cryovial was quickly thawed in a water bath at 37°C (without dissolving ice completely). Thawed cells were then carefully added dropwise to 4 mL of the recovery medium containing DNase I solution (0.5 ml), PBS (2 ml) and FBS (1.5 ml) (for every 1 mL of cell suspension) followed by incubating for 2–4 min at room temperature (RT) to dissolve any clumps completely. Cell suspension was then transferred to 1.5 mL tubes to spin down at 100 rcf for 10 min at 4°C. Supernatants were carefully removed and cells were resuspended in the remaining fluid. Pellets were combined and cell viability was assessed by trypan blue staining in a hemocytometer before cell culture. The MN cells were maintained in IMDM serum free media, supplemented with 20% BIT 9500 serum substitute, GlutaMAX (2 mM), IL3 (10 ng/mL), IL6 (10 ng/mL), IL7 (10 ng/mL), Flt3- ligand (20 ng/mL), SCF (30 ng/mL) and 10−4M 2-Mercaptoethanol (0.1 mM) and incubated at 37°C and 5% CO_2_ for at least 4 h to allow sufficient cell recovery prior to siRNA delivery studies.

### 2.3. Polymer synthesis and polymeric nanoparticle preparation for cell delivery

**Fig 1:**
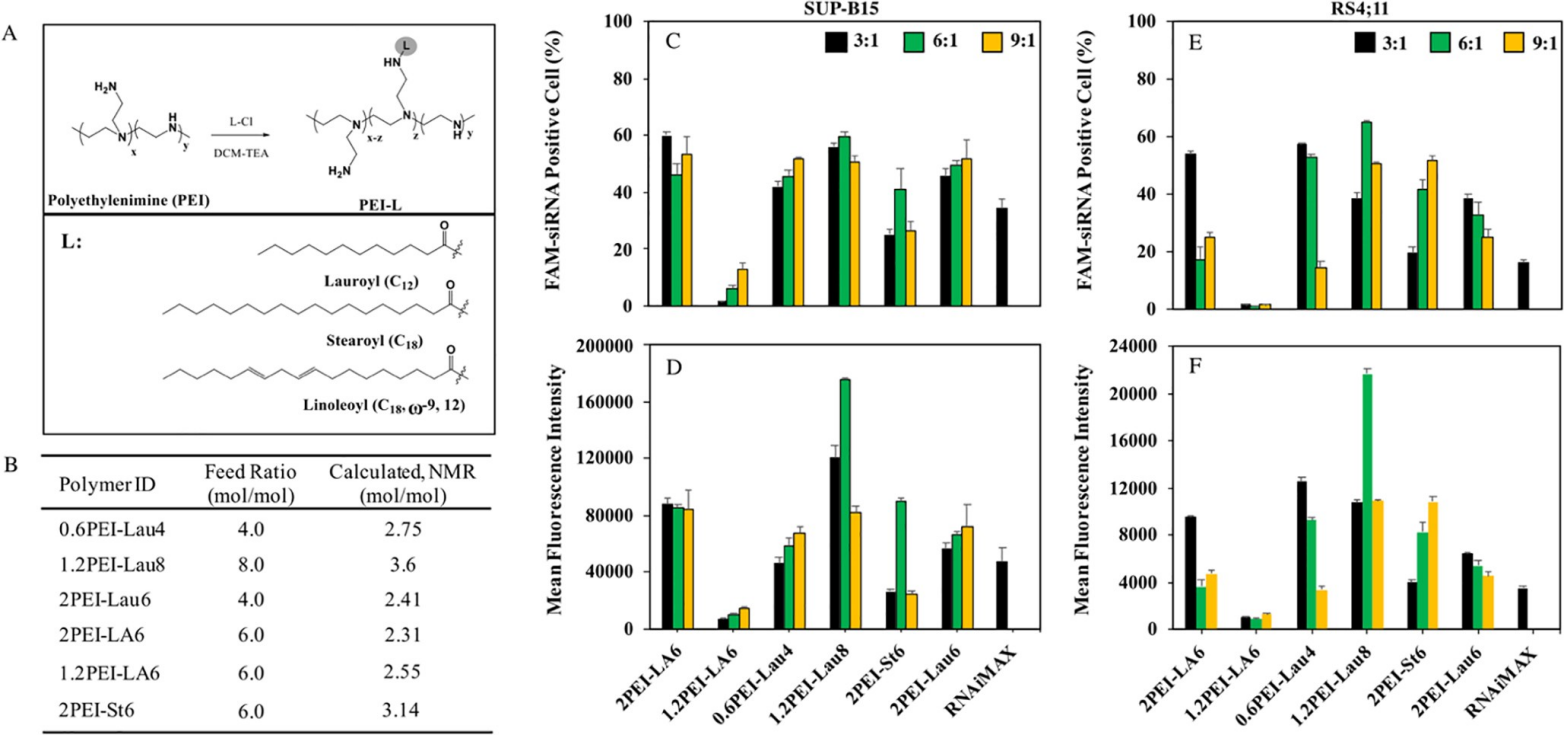
Schematic illustration of the synthesis of lipid-substituted PEIs (A). The obtained lipid substitutions as a function of lipid:polymer feed ratio during synthesis (B). (C-E) The uptake of FAM-labelled siRNA at 30 nM with polymer:siRNA ratios of 3:1, 6:1 and 9:1 after 24 hours of treatment. The percentage of cells that have taken up the siRNA are shown as FAM-positive SUP-B15 cells and RS4;11 cells (C and E, respectively) (n = 3). The mean fluorescence intensity in SUP-B15 and RS4;11 cells are also shown (D and F, respectively) (n = 3). Lipofectamine RNAiMAX was used as a reference delivery reagent at polymer:siRNA ratio of 2.5:1. FAM-siRNA positive cells and mean fluorescence were determined by flowcytometry.

Lipid-modified PEIs were synthesized according to the previously published protocols [30]. Lauric acid (Lau), linoleic acid (LA) and stearic acid (St) were used as specific lipids to modify the amines of low MW PEIs and the lipid substitution levels in final products were analyzed by ^1^H-NMR (Fig 1). Lipofectamine™ RNAiMAX Reagent was used as the lipid- based commercial reagent in all the experiments. The lipid-modified polymers and desired siRNAs were dissolved in nuclease-free water at 1 and 0.14 μg/μL, respectively. siRNA-lipid-modified polymer complexes (polymeric nanoparticles) were prepared by adding siRNA solutions to serum-free medium to get the final siRNA concentration of 60 nM in cell suspension. The polymers were then added to the siRNA solutions to give the desired polymer:siRNA weight ratios (3:1, 6:1 and 9:1, designated as R3, R6 and R9, respectively) bringing the final volume to 330 μL, followed by mixing briefly and incubating for 30 min at room temperature to allow for siRNA and polymer interaction with each other and forming complexes. Lipofectamine™ RNAiMAX Reagent-siRNA complexes were prepared at 2.5:1 lipid-to-siRNA (weight/weight) ratio (as suggested by the manufacturer) with similar siRNA concentration and incubated for 30 min at room temperature. The siRNA-polymer complexes (100 μL/well) were then added in triplicate to Falcon™ Polystyrene 48-well Microplates (Thermo Scientific, Lafayette, CO, USA) and then the cells suspended in 300 μL of culture media were added on top of the complexes. Non-treatment (NT) control groups were exposed to serum-free medium alone (no complexes), while the negative control groups were exposed to control (scrambled) siRNA/lipid-polymer nanoparticles. Cells were collected 3 and 6 days after polymeric nanoparticle treatment and analyzed for STAT5A silencing efficiency and viability.

### 2.4. siRNA uptake by flow cytometry

To determine the most effective polymeric carriers for siRNA delivery into hard to transfect leukemic cells, a library of lipid-modified PEIs was screened with FAM-labeled siRNA/polymer complexes at three polymer:siRNA ratios of 3:1, 6:1, and 9:1 and a final siRNA concentration of 30 nM in 48-well plates for 24 h. RNAiMAX was used as the reference carrier at 2.5:1 RNAiMAX:siRNA ratio. Non-labeled scrambled siRNA was used as a negative control. After 24 hours of incubation time, cells were collected in microcentrifuge tubes and centrifuged (1800 rpm for 8 min), washed twice with HBSS (pH 7.4) and re-suspended in formalin at a final concentration of 3.7% in HBSS. The uptake efficiency of complexes was quantified by a BD Accuri^TM^ C6 Plus flow cytometer using the FL1 channel (10,000 events per sample) and calibrating the instrument so that autofluorescence of the negative control (untreated cells) gave ~1% of the total cell population as the background. The mean fluorescence of the recovered cell population and the percentage of cells showing FAM-fluorescence were measured to evaluate the siRNA delivery in cells.

### 2.5. Reverse transcription quantitative polymerase chain reaction (qPCR) for assessing the silencing activity of siRNAs

We used qPCR analysis for investigation of siRNA silencing since assessing protein silencing (by western blots) was going to require 3–4 fold increased cell mass, which was not available for most primary patient samples. To explore the silencing effect of desired siRNAs, the leukemic cells were transfected in 6-well plates with complexes prepared with STAT5A, BCR-ABL and control siRNAs at a 60 nM siRNA concentration and the effective polymers at 3:1 and 6:1 polymer:siRNA weight ratios for 3 and 6 days. After the incubation time, cells were collected in microcentrifuge tubes, spun down, and total RNA was extracted using TRIzol reagent following manufacturer’s instructions. The quantity and integrity of total extracted RNA was then assessed by optical density measurement (A260/A280 ratio) using spectrophotometer (GE Nanovue). One microgram of total RNA was converted into cDNA using SensiFAST cDNA Synthesis Kit according to manufacturer’s recommendations. Finally, using a StepOnePlus real-time PCR system (Applied Biosystems, Foster City, CA), real-time PCR analysis with 2× SYBR green master mix and ROX (MAF Center, University of Alberta) was performed to follow the fluorescence intensity. Specific primers applied to detect the expression levels of human beta-actin (housekeeping endogenous gene) (forward: 5′-GCGAGAAGATGACCCAGAT-3′; reverse: 5′-CCAGTGGTACGGCCAGA-3′), STAT5A (forward: 5′-CCGACGGGACCTTCTTGTTG-3′; reverse: 5′-TGCGTTCCGGGGAGTCAAAC-3′), and BCR-ABL (forward: 5′-CATTCCGCTGACCATCAATAA G-3′; reverse: 5′-GATGCTACTGGCCGCTGAAG-3′) were designed by the NCBI Primer-BLAST and supplied by IDT. A 10 μL volume containing 5 μL of 2× master mix SYBR Green, 1 μL of 10 μM forward primer, 1 μL of 10 μM reverse primer and 3 μL of cDNA template (5 ng/μL) for each sample (three independent biological replicates) were transferred to a Fast Optical 96-well plate. The cDNA template was omitted from qPCR reaction as a negative control in qPCR. The amplification cycle consists of heating the reaction mixtures for 5 min at 95°C before going through 40 cycles of a denaturation step (15 s at 95°C) and an annealing/elongation step (1 min at 65°C). The level of target gene expression was determined by 2^−ΔΔCT^ method using the non-treatment groups as the calibrator. Target gene CT values were normalized against beta-actin CT values and the results were reported as the relative quantity of transcripts.

### 2.6. MTT assay to evaluate inhibition of cell growth following STAT5A silencing

The efficacy of STAT5A silencing on cell growth inhibition was investigated using MTT assay. In 48-well plates, 45,000 RS4;11 cells, 50,000 SUP-B15 cells and 2 × 10^5^ ALL primary cells suspended in 300 μL of culture medium, were transfected with the indicated siRNA-polymer complexes for 3 and 6 days. At desired time points, MTT solution (5 mg/mL) was added to the wells to give a final concentration of 1 mg/mL and the cells were incubated for 2 h further at 37°C, after which the cells were collected by centrifugation and the medium was removed. The formed formazan crystals were then dissolved with 100 μL of DMSO and the absorbance of the wells was measured with an ELx800 Universal Microplate Reader (BioTek Instruments, VT, USA) at 570 nm. The cell viability percentage was calculated as follows: 100% × (absorbance of polymeric nanoparticle treated cells/absorbance of untreated cells).

### 2.7. Trypan blue exclusion assay for cell viability

To evaluate cell growth inhibition, cells were transfected for 3 and 6 days with complexes. After incubation, trypan blue exclusion assay was performed by mixing 20 μL of cell suspension with 20 μL of trypan blue dye and injecting into a hemocytometer. The numbers of viable cells that exclude the dye were counted and cell concentration (the number of viable cells per ml) was then calculated.

### 2.8. Colony-forming cell (CFC) assay

To assess the inhibition of cell proliferation induced by siRNA/polymer complexes, leukemic cells were transfected with complexes at a polymer:siRNA ratio of 6:1 and final siRNA concentration of 60 nM. 24 hours after the transfection, trypan blue exclusion staining was used to count the viable cells in a hemocytometer. 400 RS4;11 and SUP-B15 cells, or 15,000 ALL primary cells were then mixed in 0.5 mL of methylcellulose media and seeded in the center wells of the 24-well plates. For the CFC assay of ALL primary cells, human methylcellulose-enriched media supplemented with final recombinant human EPO (3 IU/mL), recombinant human IL-6 (20 ng/mL), recombinant human IL-3 (20 ng/mL), recombinant human GM-CSF (20 ng/mL), recombinant human G- CSF (20 ng/mL), recombinant human SCF (50 ng/mL), 2-Mercaptoethanol (5 x 10^−5^ M), L-Glutamine (2mM), 2% Bovine Serum Albumin (BSA), 25% FBS, and 1.4% IMDM was applied while for ALL cell lines, human methylcellulose-based media containing 1.4% IMDM, 25% FBS, 2% BSA, 2-Mercaptoethanol (5 x 10^−5^ M) and L-Glutamine (2mM) was used. The total number of colonies were observed and counted with optic microscopy after 14 days of incubation at 37°C in a fully humidified incubator with 5% CO_2_.

### 2.9. Combinational siRNA therapy

Combinational siRNA delivery was performed in ALL BCR-ABL positive primary cells using STAT5A and BCR-ABL siRNAs at total siRNA concentration of 60 nM (30 nM each) with a 6:1 polymer:siRNA weight ratio. Individual STAT5A or BCR-ABL siRNA at total 30 and 60 nM concentrations with 6:1 polymer:siRNA weight ratio was delivered by 1.2PEI-Lau8 polymer in ALL patient cells as well. The efficacy of combinational siRNA therapy to inhibit cell growth and induce gene silencing was investigated after 72 hours by MTT assay, cell counting, and qPCR as described above, respectively. Scrambled siRNA was used as the negative control in the experiments.

### 2.10. Statistical analysis

All results were reported as mean ± standard deviation (SD). Statistical analysis was performed by unpaired Student’s t test, where significantly different groups were determined by an asterisk (*) in figures. A value of p ≤ 0.05 was considered statistically significant and it was defined by comparing specific siRNA/polymer-treated groups to that of control siRNA/polymer-treated groups.

## 3. Results

### 3.1. siRNA uptake in RS4;11 and SUP-B15 cells

We evaluated and compared the potential of a library of lipid-substituted low MW PEI derivatives to transfect ALL cells. The scheme for synthesis of lipid-substituted PEIs and lipid substitution levels as a function of feed ratio are displayed in Fig 1A and 1B, respectively. The level of substitution ranged between 2 and 4 lipids/PEI and the MW of PEI backbone did not affect the level of substation among the tested carriers. In SUP-B15 cells, four polymer groups exhibited high FAM-siRNA uptake at most ratios (Fig 1C). A similar observation was made in RS4;11 cells (Fig 1E), among which 1.2PEI-Lau8 was the highest, with an overall range between 40–50% FAM positive cells. Considering the mean fluorescence intensity in both the cell lines, 1.2PEI-Lau8 at ratio 6:1 gave the highest uptake which was significantly (p≤ 0.005) different when compared to other ratios (3:1 and 9:1) (Fig 1D and 1F). The commercial reagent RNAiMAX gave lower FAM-siRNA positive cells and mean fluorescence intensity compared to the polymeric delivery systems.

### 3.2. STAT5A gene knockdown

**Fig 2:**
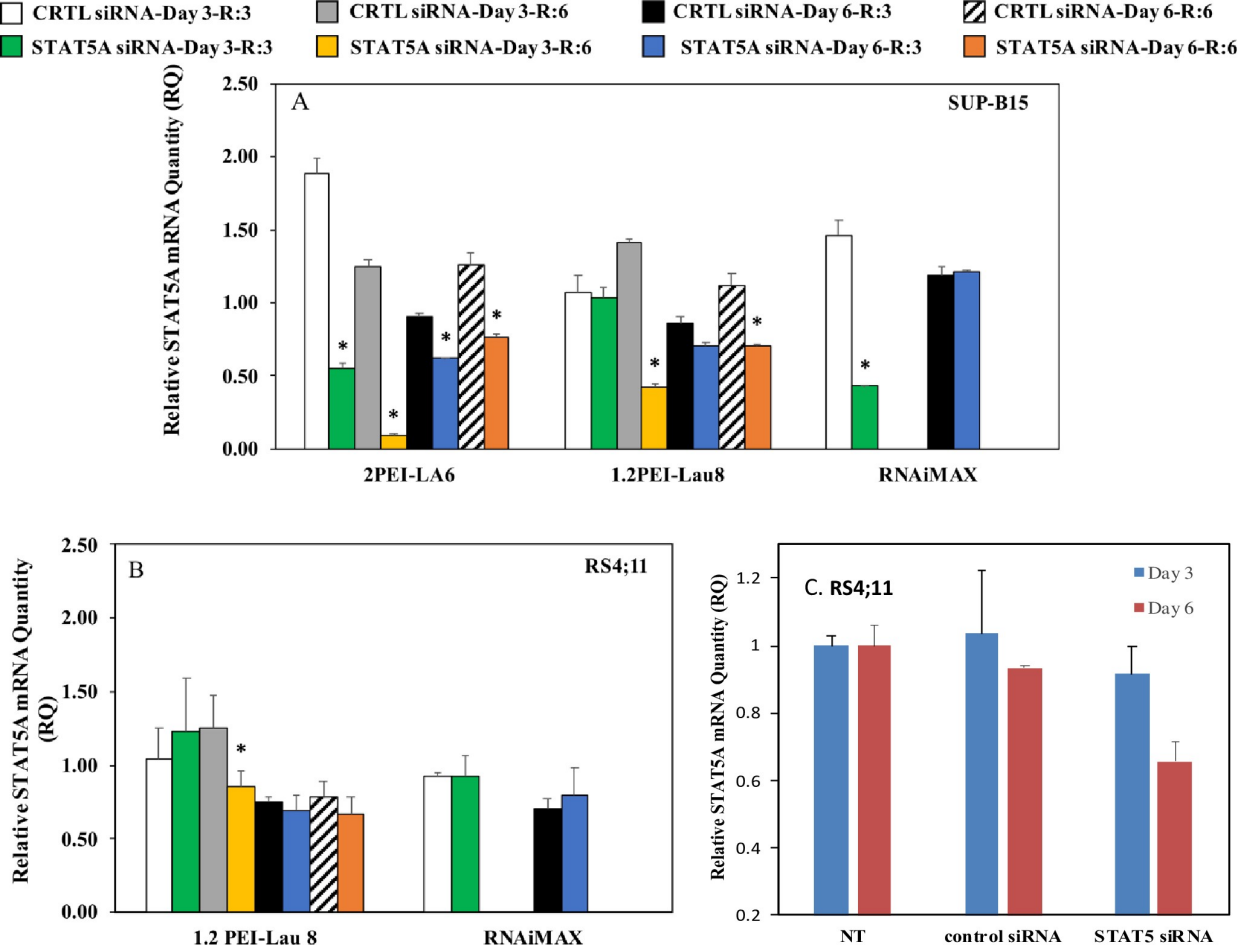
STAT5A mRNA levels after siRNA treatment. The relative STAT5A mRNA levels (relative to β-actin as an internal control) were quantified through qPCR. SUP-B15 (A) and RS4;11 (B and C) cells were treated with 60 nM of STAT5A/CTRL siRNAs for 3 and 6 days and RQ of mRNA are plotted relative to non-treatment group. PEI-Lau8 and PEI-LA6 were used in B and C, respectively, while Lipofectamine RNAiMAX was used as a reference delivery reagent in B. (n = 3) *p ≤ 0.05 vs. Control siRNA.

STAT5A silencing was evaluated at the mRNA level using qPCR. In SUP-B15 cells, two of the polymer groups that showed high siRNA uptake, 1.2PEI-Lau8 and 2PEI-LA6, were chosen to evaluate gene silencing. The 1.2PEI-Lau8 polymer was able to silence 70% and 36% of STAT5A gene expression at ratio 6:1 on both day 3 and day 6, respectively, whereas 2PEI-LA6 at the same conditions, silenced 92%, at ratio 6:1 on day 3 compared to the CTRL siRNA groups (Fig 2A). In RS4;11 cells, 1.2PEI-Lau8 was effective in lowering the STAT5A gene expression (32%) only at ratio 6:1/day 3 (Fig 2B). RNAiMAX exhibited 71% silencing, only on day 3 in SUP-B15 cells in comparison with the CTRL siRNA groups (Fig 2A). With PEI-LA6 and RS4;11 cells (Fig 2C), silencing was assessed only at ratio 3 with control siRNA and STAT5 siRNA; significant silencing (~28%) was evident on day 6.

### 3.3. Cell growth inhibition by STAT5A silencing

In the cell viability assessment, the 1.2PEI-Lau8/STAT5A complexes were able to inhibit the growth by 39% in SUP-B15 cells on day 3 and 24–27% in RS4;11 cells on day 3 and day 6 compared to polymer/CTRL siRNA groups (p≤0.05; S1A and S1B Fig). On the other hand, 2PEI-LA6/STAT5A complexes could only exhibit minimal decrease in the cell viability in both SUP-B15 cells (20%; S1A Fig) and RS4;11 cells (15–18%; S1B Fig) irrespective of the time points.

**Fig 3:**
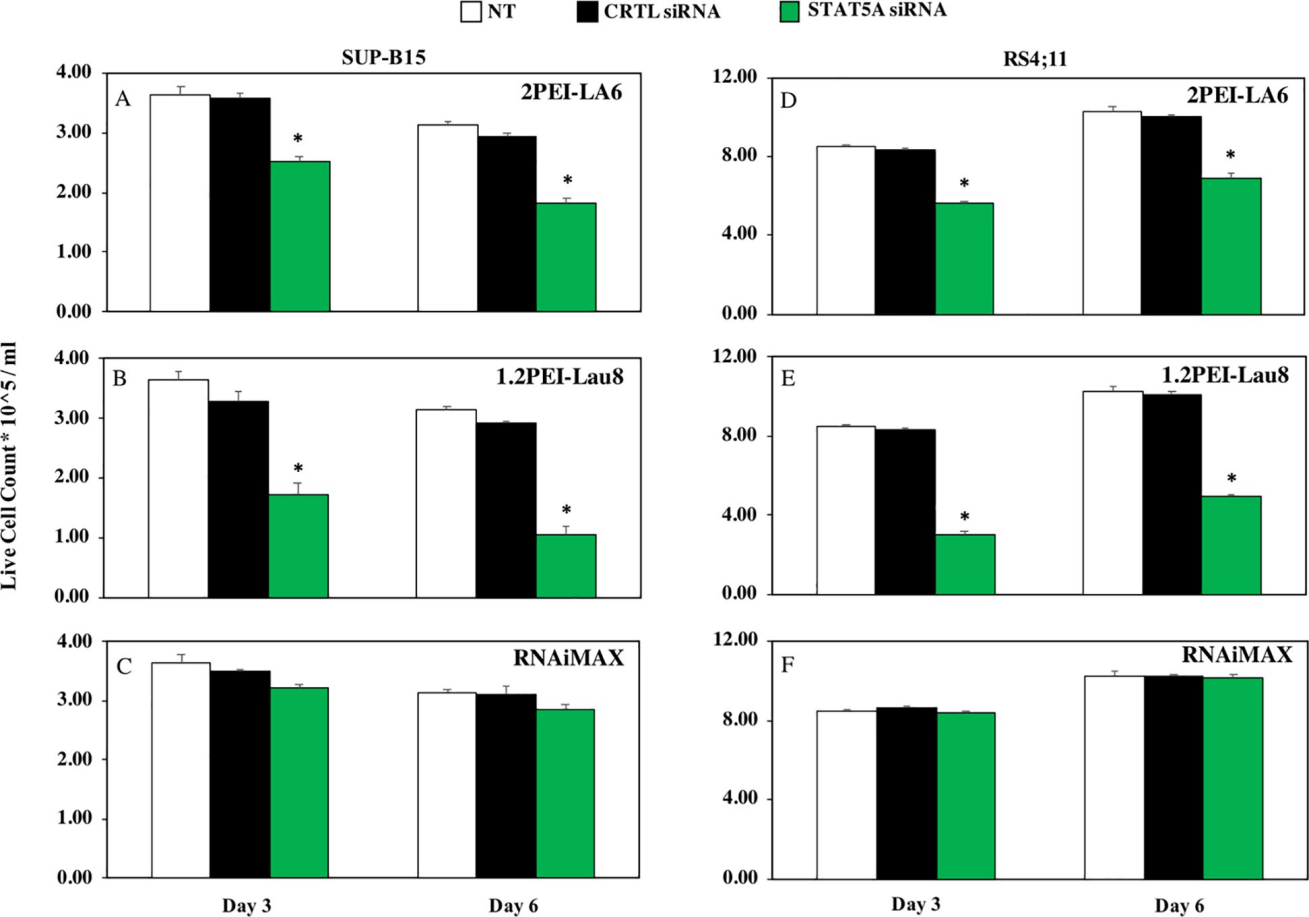
The growth inhibition of cells by complexes. Cells were transfected with CTRL/STAT5A siRNA complexes and live cells were counted by trypan blue exclusion assay after 3 and 6 days of treatment. Y-axis indicates the live cell count (x 10^5) per ml. Live cell counts in SUP-B15 and RS4;11 cells using 2PEI-LA6 (A and D), 1.2PEI-Lau 8 (B and E) polymers along with RNAiMAX (C and F) are indicated at two time points and values are shown as means ± SD; (n = 3) *p ≤ 0.05 versus control siRNA.

Using trypan blue exclusion assay in both cell lines, 2PEI-LA6/STAT5A siRNA complexes were able to exhibit a 1.5-fold significant decrease in the live cell count compared with CTRL siRNA complexes on both time points (p≤0.05; Fig 3A and 3D). The leading 1.2PEI-Lau8/STAT5A siRNA complexes showed a stronger effect on both cell lines with 1.7 and 2.7-fold decrease in live SUP-B15 cells and 2 and 2.7-fold decrease in RS4;11 cells on day 3 and day 6, respectively (p≤0.05; Fig 3B and 3E).

### 3.4. Inhibition of cell growth by CFC assay following STAT5A knockdown

**Fig 4:**
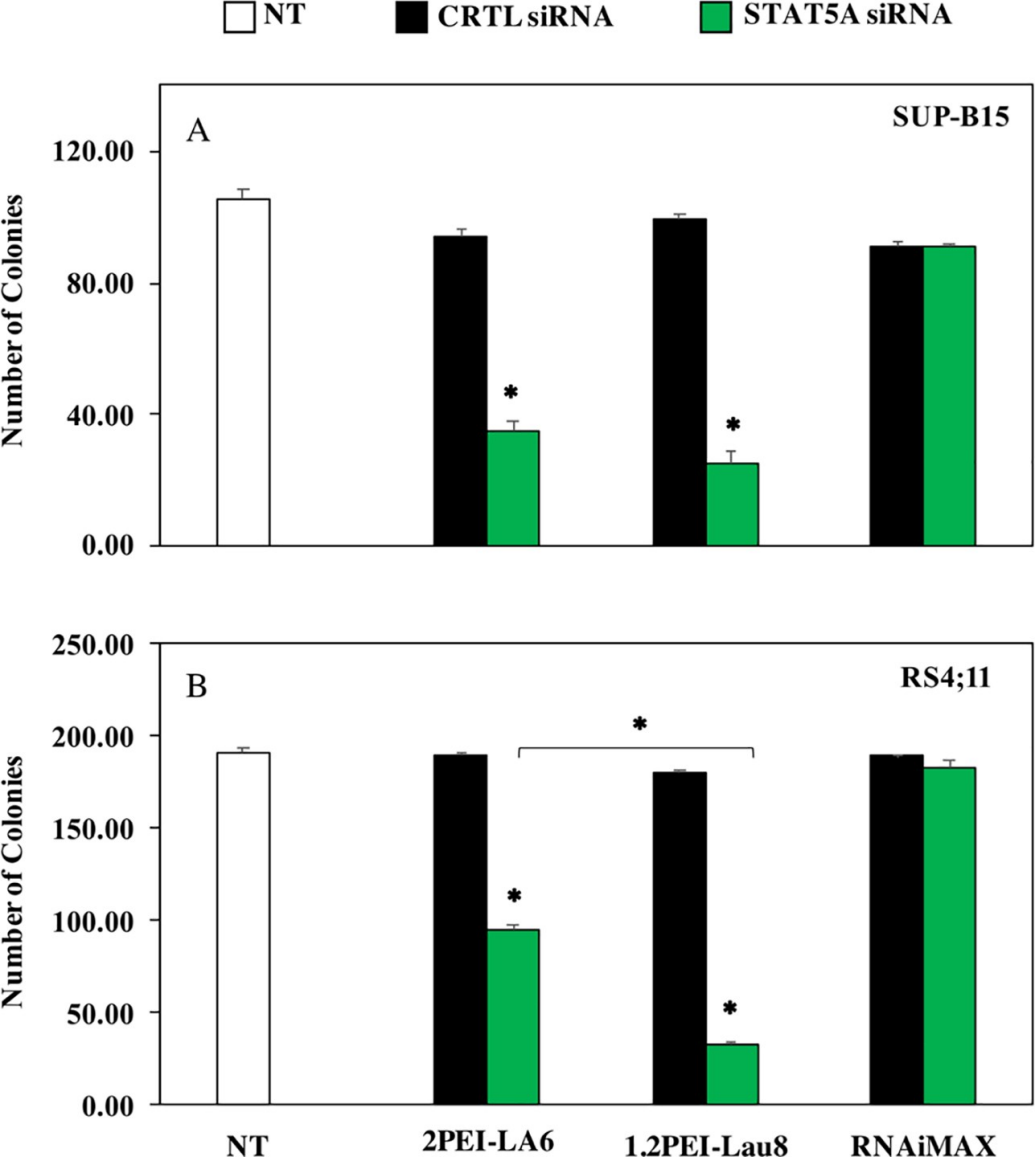
Effects of transfection with CTRL/STAT5A siRNA/polymer complexes on colony formation in leukemic cell lines. Colony counts were performed two weeks after treatment at concentration of 60 nM and at polymer:siRNA ratio of 6, (RNAiMAX:siRNA ratio: 2.5:1). (A) Total number of colonies formed by SUP-B15 cells (A) and RS4;11 cells (B). (n = 3) *: p ≤ 0.05.

In CFC assay, 2PEI-LA6 polymer was able to show 2.7-fold and 2-fold significant decrease in colony counts in SUP-B15 and RS4;11 cells, respectively, compared to the control siRNA group (p≤0.05; Fig 4A and 4B). Moreover, a 3.9-fold and 5.4-fold marked reduction in the colony formation was induced by 1.2PEI-Lau8 in SUP-B15 and RS4;11 cells, respectively (p≤0.05; Fig 4A and 4B).

### 3.5. Functional outcome of STAT5A silencing in ALL primary cells

**Fig 5:**
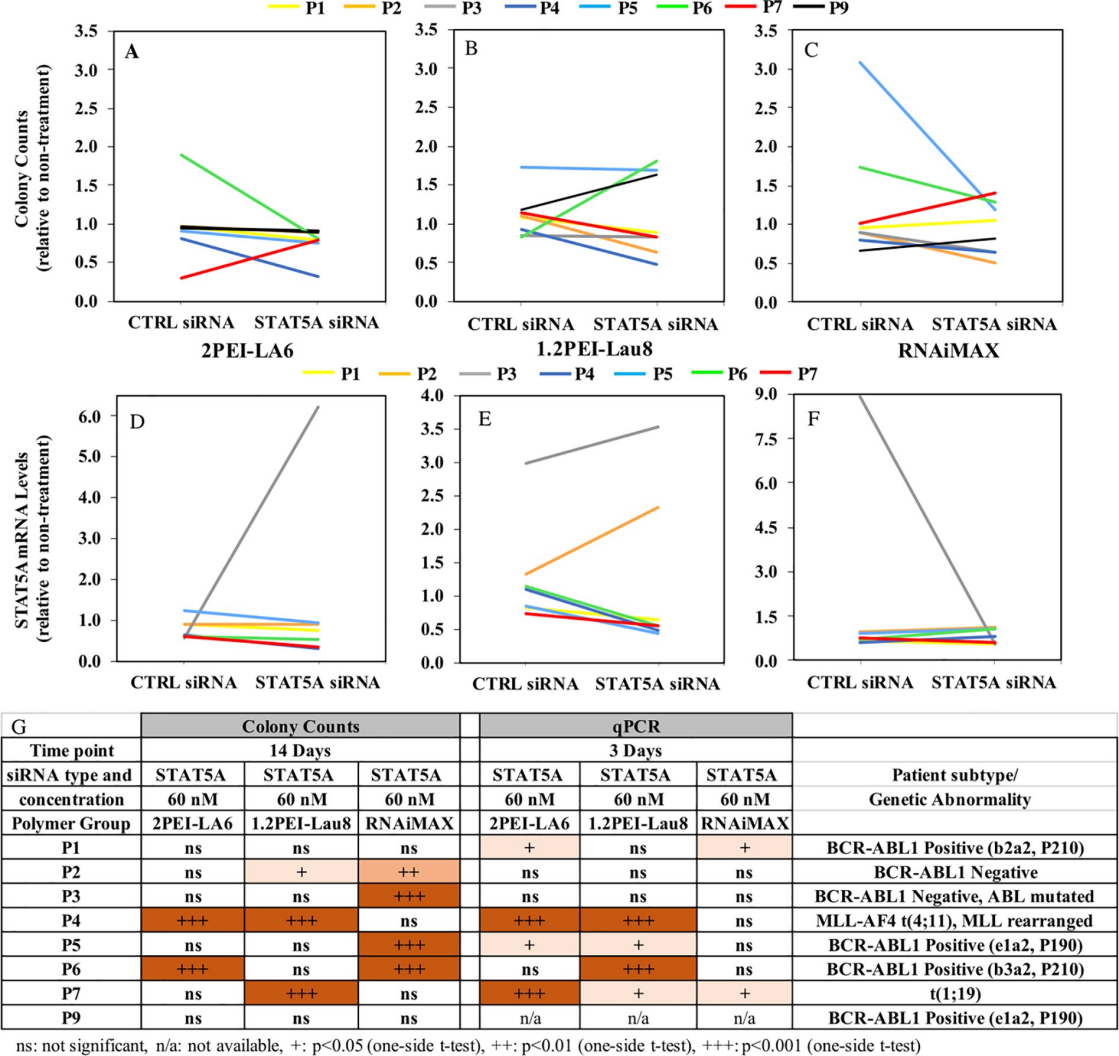
Effects of treatment with STAT5A siRNA/polymer complexes on colony formation (A, B and C) and STAT5A mRNA expression (D, E and F) in ALL primary cells. Colony counts were performed two weeks after transfection at a siRNA concentration of 60 nM and polymer:siRNA ratio of 6 and RNAiMAX:siRNA ratio of 2.5 for one day. STAT5A mRNA levels were assessed by qPCR after 3 days of transfection on frozen MN patient samples (n = 3). (G) Summary of statistical analysis for the results from qPCR and CFC assays.

**Fig 6:**
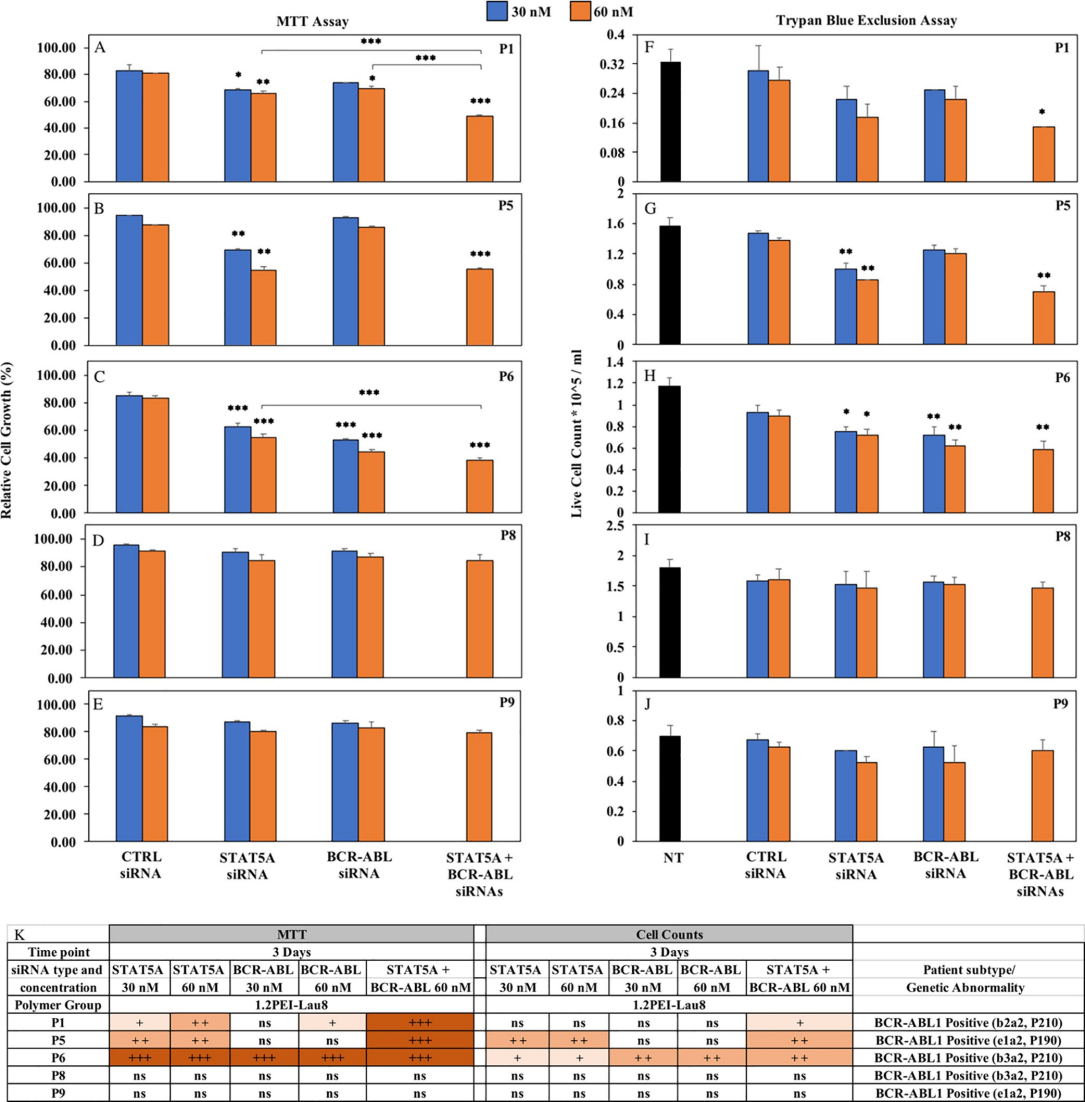
Effect of siRNA/polymer complexes on proliferation of BCR-ABL positive patient cells explored by MTT assay (A-E) and trypan blue exclusion assay (F-J). Cells were treated with 1.2PEI-Lau8/siRNA ratio of 6:1 and 30 nM and 60 nM of Control, STAT5A and BCR-ABL siRNAs for 3 days. Cell growth inhibition was assessed by MTT Assay and expressed relative to non-treated cells (taken as 100%). In addition, live cells were counted by trypan blue exclusion assay. Y-axis indicates the live cell count (x 10^5) per ml. Values are shown as means ± SD; (n = 3) *: p<0.05, **: p<0.01, ***: p<0.001 versus control siRNA. (K). Summary of significant differences in MTT and cell counts in BCR-ABL positive patient samples.

The inhibition in cell growth and proliferation observed in cell lines was further investigated in ALL patient cells by the CFC assay (Fig 5A, 5B and 5C) and qPCR (gene silencing) (Fig 5D, 5E and 5F). Some of the frozen patient samples (P8 and P10) had limited number of viable cells due to the poor cell recovery in the thawing process; therefore, some assays could not be performed (denoted as n/a [not available] in Figs 5G and 6E). In 2PEI-LA6/STAT5A complex treatment groups, two samples, P4 and P6, showed a significant decrease in the colony counts in comparison with their control siRNA groups (P4: 61.7% and P6: 56.6%, p≤0.05; Fig 5A and 5G). With 1.2PEI-Lau8/STAT5A siRNA complexes, the colony counts decreased significantly by 42.3%, 48.39 and 28% in P2, P4 and P7, respectively (p≤0.05; Fig 5B and 5G).

Based on the qPCR assay, 2PEI-LA6 complex treatment significantly decreased STAT5A mRNA levels in four patient samples (P1, P4, P5 and P7) by 18%, 51.6%, 24.2% and 44.8%, respectively, compared to their control siRNA groups (p≤0.05; Fig 5D and 5G). In the 1.2PEI-Lau8 treatment groups, there was a marked reduction in STAT5A mRNA levels in P4, P5, P6 and P7 by 57.3%, 47.6%, 51.3% and 23.6%, respectively (p≤0.05; Fig 5E and 5G). STAT5A silencing with RNAiMAX was evident in P1 (17.6%) and P7 (22.8%) in comparison with control siRNA groups (p≤0.05; Fig 5F and 5G).

### 3.6. Combinational silencing of BCR-ABL and STAT5A in BCR-ABL positive ALL patient cells

Among the 10 patient samples available in this study, 5 samples were BCR-ABL positive and, therefore, the extent of both STAT5A and BCR-ABL gene silencing and cell growth inhibition was investigated by qPCR, MTT and trypan blue exclusion assays. The STAT5A mRNA levels significantly decreased with 30 nM of STAT5A siRNA (72.7%) as well as with 30 nM of BCR-ABL siRNA (69.4%) only in the P5 sample, in comparison with the control siRNA group (S2A and S2E Fig; p ≤0.05). The combination of STAT5A (30 nM) and BCR-ABL (30 nM) siRNAs resulted in the downregulation of STAT5A gene in samples P1 (16.2%) and P5 (67.4%) compared to their control siRNA groups (S2B and S2E Fig; p ≤0.05).

Using the MTT assay, STAT5A siRNA at both 30 nM and 60 nM showed a significant cell growth inhibition in 3 out of 5 patient samples compared to their respective control groups; P1, P5 and P6 (Fig 6A, 6B and 6C; p ≤0.05). By trypan blue exclusion assay, the live cell number was significantly reduced compared to their respective controls in 2 of these samples, P5 and P6, at both 30 nM and 60 nM (Fig 6G and 6H; p ≤0.05). After transfected with BCR-ABL siRNA, cell growth was markedly inhibited in only one patient sample at 30 nM and 2 patient samples (P1 and P6) at 60 nM (Fig 6A, 6B and 6C; p ≤0.05). By trypan blue exclusion, transfection with BCR-ABL siRNA led to marked decrease in live cell counts in only one patient sample (P6) at 30 nM and 60 nM siRNA (Fig 6H). The combination of STAT5A and BCR-ABL siRNAs resulted in a significant reduction in cell growth in comparison with their control siRNA groups in 3 samples (P1: 32.6%, P5: 31.6%, P6: 45.2%) (Fig 6A, 6B and 6C; p ≤0.05). This correlated with a significant drop in the live cell number by MTT assay in 3 patients: P1: 46.4%, P5: 49.3% and P6: 35.6%, (Fig 6F, 6G and 6H).

In BCR-ABL negative ALL primary cells, STAT5A siRNA decreased the cell viability by MTT assay in 3 out of 5 patient samples compared to their respective control groups (P2, P4 and P7) at both 30 nM and 60 nM. (S3A, S3C and S3D Fig). By trypan blue assay, the live cell number significantly reduced with STAT5A siRNA 30 nM in the same 3 patient samples (S3F, S3H and S3I Fig), and in samples P2 and P7 at 60 nM (S3F and S3I Fig).

## 4. Discussion

RNAi has become a promising therapeutic approach for cancer and its success can be observed at various stages of clinical trials in different malignancies [31–33]. In ALL, various targets have been successfully silenced by RNAi, such as Polo-like kinase 1 (Plk1), CD22ΔE12, CSF1R, JAK1 and FLT3 [34–36], all of which highlights the versatility of using RNAi for ALL therapy. In this study, we have shown the feasibility of siRNA-mediated STAT5A silencing in ALL cell lines SUP-B15 and RS4;11 as well as ALL patient cells from different donors, using 2 lipid modified PEIs. We have demonstrated a strong reduction in mRNA levels which led to a sharp decrease in live cell counts and colony formation in ALL cell lines. The 1.2PEI-Lau8 polymer emerged as the leading candidate from this screen. The same polymer, when previously applied in breast cancer studies, only exhibited moderate performance for siRNA delivery [37], indicating the need to tailor delivery systems for specific cell phenotypes. The other effective polymer 2PEI-LA6 was successful in other cancer types such as AML, CML and breast cancer [25, 37–39], indicating a more universal applicability of this delivery system. The smaller LA derivative of PEI (1.2PEI-LA6) did not demonstrate effective siRNA delivery in both cell lines, presumably due to smaller size and relatively less efficient binding to siRNA; however, in previous reports, the same polymer was able to effectively deliver siRNA to MDA-MB-231 and MCF7 breast cancer cells. This emphasizes that different polymer formulations might be required for different cell types and the functionality of the same polymer could vary among various cell lines owing to the different cell properties [37, 40]. Other studies showed that the efficiency of these polymers was related to their physiochemical characteristics including the optimal degree of lipid substitution, type of the lipid substituent, MW, charge and size of the siRNA nanoparticles [28, 30, 41], which were not repeated in this study. The reason(s) for low efficacy observed with some delivery agents was not explored here, but could be attributed to low binding or poor complexation ability with siRNA leading to reduced entry into the cells, weak interactions of complexes with cell membrane, unsuccessful endosomal escape leading to lysosomal degradation or tight binding resulting in the lack of siRNA dissociation inside the cytoplasm [42, 43].

The siRNA uptake for SUP-B15 was ~10-fold higher to that observed in RS4;11 cells (e.g., 1.2PEI-Lau8 at ratio 6:1 gave uptake of ~200,000 au in SUP-B15 vs. ~24,000 in RS4;11 cells; Fig 1B and 1D). This difference was reflected in the STAT5A mRNA reduction levels, where both polymers showed strong silencing in SUP-B15, whereas the effect was attenuated in RS4;11 (Fig 2). This supports previous reports on the correlation between uptake and silencing efficacy [26, 39, 40]. STAT5A silencing efficiency of 2PEI-LA6 was considerably higher than that of 1.2PEI-Lau8 at ratio 6:1 in SUBP-B15 even though the latter polymer showed significantly higher uptake. This could be attributed to a better siRNA release in the cytoplasm with 2PEI-LA6 than 1.2PEI-Lau8. 2PEI-LA6 did not exhibit any difference in uptake with SUP-B15 at different ratios; however, the silencing was much stronger at ratio 6:1, which could point to a better siRNA release in the cytoplasm at this particular ratio. 2PEI with LA substitution has been previously shown a tendency to dissociate more rapidly due to its large linoleoyl in studies with cutaneous T cell lymphoma and AML cells [38, 44]. This emphasizes the importance of releasing free siRNA in the cytoplasm for incorporation into RISC to induce the cleavage and degradation of target mRNA. Although the details of trafficking was not investigated here, a recent study by our group indicated the effective internalization of siRNA/PEI-LA complexes by confocal microscopy in K562 cells and CML primary cells [39]. 1.2PEI-Lau8 showed successful silencing at ratio 6:1 that was expected from its highest siRNA uptake at this ratio. The same polymer did not reveal any reduction of STAT5A mRNA levels at ratio 3:1, even though, its uptake was considerably higher, which could be due to low siRNA loading ability at this ratio. In RS4;11, 1.2PEI-Lau8 showed significant reduction of STAT5A mRNA only on day 3 at ratio 6:1 which was consistent with the uptake results. It is noteworthy that the polymeric delivery systems were able to sustain STAT5A mRNA silencing up to day 6, whereas RNAiMAX could not exhibit this efficacy. Moreover, the safety profile of our polymeric delivery systems was previously reported using normal human skin fibroblasts and the effect of treatments was negligible in these cells [45].

Subsequent studies in both cell lines demonstrated that these two PEI’s complexed to STAT5A siRNA were able to effectively inhibit cell growth, as measured by MTT assay, and to reduce cell viability by trypan blue exclusion Figs 3 and 4). This highlights the important role of STAT5A in the survival of these ALL cells. The reduction of live cells with 1.2PEI-Lau8 was more evident at both time points (with stronger effect on day 6) compared to 2PEI-LA6. The live cell count appears to slightly decrease on day 6 for SUP-B15, whereas in RS4;11 cells, the day 6 counts were higher irrespective of the carrier used. This observation could be due to the differences in the doubling time of 60 vs. 35 hours for SUP-B15 vs. RS4;11 cells, respectively [46].

A recent study also demonstrated high siRNA uptake, marked reduction of STAT5A mRNA levels as well as increase in cell death using lipid-modified PEIs in breast cancer cell lines, which is consistent with our findings here and supports the efficacy of our delivery system [47]. The different siRNA silencing efficiencies between the ALL cell lines could be explained by differences in endosomal processing pathway or endocytic activities and also different expression levels of STAT5A in the target cells, as reported previously [3, 48, 49]. Both 2PEI-LA6 and 1.2PEI-Lau8 displayed a significantly stronger colony inhibition than the cell viability assays; the results were most profound with 1.2PEI-Lau8, especially in RS4;11 cells (p ≤0.05). These outcomes highlight the high efficacy of the polymer/siRNA complexes as well as the key role of STAT5A in cell proliferation. In addition, our results corroborate other studies on STAT5 as a therapeutic target in ALL [3, 50].

With respect to patient samples, we observed significant variability in the response to the siRNA treatments. Five of 8 samples showed significant STAT5 silencing with at least one of the delivery systems (Fig 5). Similarly, colony formation was significantly reduced in 6 of 8 samples with at least one polymeric construct (Fig 5). With our leading polymeric delivery system, 1.2PEI-Lau8, 4 of 8 samples showed significant reduction of STAT5A levels based on qPCR assessment and 3 of 8 showed a reduction in colony formation. This emphasizes the importance of individualizing such treatment approaches. The expression levels of target genes might have played an important role; low STAT5 expression, strong upregulation or the presence of redundant signaling pathways could have hindered the silencing effect, thereby inhibiting the ability to affect the cell growth and proliferation [51]. We also could not exclude variability in cellular uptake between different patient samples.

In BCR-ABL positive samples, silencing using STAT5A siRNA (60 nM) was successful in one case (P1), accompanied by a reduction in cell growth by MTT assay; however, there were no changes in colony and cell counts. Notably, though BCR-ABL silencing was not evident by itself, the combination of siRNA to both BCR-ABL + STAT5A resulted a statistically significant reduction in cell growth in two samples, consistent with the observation that STAT5 is a downstream target of BCR-ABL for phosphorylation [52]. However, in other samples no downregulation was observed at the concentrations used. As we did not measure cellular uptake in patient samples due to limitations in cell numbers, the possibility that uptake may have been suboptimal in those cases could not be excluded.

The P4 sample, with an MLL rearrangement, consistently gave significant reduction in STAT5A mRNA and colony counts with 2PEI-LA6 and 1.2PEI-Lau8. Furthermore, cell growth was inhibited with STAT5A siRNA in MTT and cell counts (at 30 nM siRNA). Some studies have found that, in MLL-rearranged ALL, STAT5 is persistently activated by FLT3-ITD which in turn induces the expression of its downstream target [53, 54]. Due to presumably sustained activation of STAT5 in this MLL-rearranged ALL, STAT5A silencing was more beneficial and promoted proliferation inhibition, possibly by downregulating PIM-2 [54, 55]. It was also shown that a potent STAT5 SH2 domain inhibitor, AC-4–130, was beneficial against FLT3-ITD-mediated activation of STAT5 for supressing proliferation and colony formation in FLT3-ITD^+^ AML primary cells *in vitro* and *in vivo* [55]. The P7 sample, which had translocation t(1;19,) gave a strong reduction in STAT5A mRNA levels with all polymer/siRNA complexes, with a decrease in colony counts using 1.2PEI-Lau8, accompanied by suppression of cell growth by. The role of STAT5-mediated signaling in ALL patients with t(1;19) has not been investigated and our findings suggest that STAT5A may be important in this subset of ALL patients.

Other reasons for the heterogeneity of responses may include variations in qPCR assay conditions [56, 57] and variable time to observe responses; a more frequent analysis of mRNA levels might better link changes in target mRNA levels to growth assessments. While differences between the MTT and cell count results observed in some patients (e.g., P1, P2 and P4) could be due to alterations of cell metabolism (i.e., up- or down-regulation of enzymatic activity), distinctly different from membrane integrity events [58], these differences are more likely due to underlying differences in STAT5 related signaling events. Since the patient cells exhibited different cytogenetics and likely possess different genetic and signalling profiles, it is not surprising that STAT5A siRNA treatments did not lead to uniform responses in all patient samples. Such response heterogeneity also exists with current drugs for ALL patients. These findings highlight the importance of developing individual approaches for ALL treatment [59–61], whether it is conventional drug or siRNA based.

In conclusion, we have demonstrated, for the first time, the successful delivery of STAT5A siRNA via polymeric carriers into ALL cell lines, which was accompanied by marked inhibition of STAT5A expression (confirmed only at mRNA levels) and reductions in cell viability and proliferation. This outcome was also reflected in some patient-derived ALL primary cells, where STAT5A knockdown decreased the total number of colonies and inhibited cell growth. These data support the importance of STAT5A as a potential therapeutic target in ALL as well as the potential role of this polymer-based delivery system. It was clear that the extent of lipid modification in polymers and complex formulation details (in particular polymer:siRNA ratio) are important and requires attention for the final efficacy of gene silencing. Additional primary ALL cells should be evaluated to more thoroughly investigate the response heterogeneity, and to correlate the responses with cell uptake, baseline protein expression and genetic profiles. Effects on normal bone marrow and peripheral mononuclear cells should also be evaluated to get a better sense of undesired effects of STAT5 silencing. Furthermore, strategies using chemotherapeutic agents and tyrosine kinase inhibitors combined with STAT5A siRNA should be explored, both *in vitro* and in animal models, which could potentially improve the efficacy of existing ALL therapies and help circumvent drug resistance.

## Supporting information

S1 Fig Effect of siRNA/polymer complexes on proliferation of SUP-B15 (A) and RS4;11 (B) cells. Cells were treated with polymer/siRNA ratio of 6:1 and 60 nM of Control/STAT5A siRNA complexes for 3 and 6 days and cell growth inhibition was assessed by the MTT Assay and expressed relative to non-treated cells (taken as 100%). The data are the mean ± SD. (n = 3) *p ≤ 0.01 compared with the complexes with Control siRNA.
(PDF)

S2 Fig Effects of treatment with STAT5A and BCR-ABL siRNA/polymer complexes on STAT5A (A and B) and BCR-ABL (C and D) mRNA expression in ALL primary cells. mRNA levels were assessed 3 days after transfection at siRNA concentrations of 30 and 60 nM and 1.2PEI-Lau8 polymer:siRNA ratio of 6 by qPCR. (n = 3) (E) Summary of statistical analysis for the results from qPCR results of combination treatment.
(PDF)

S3 Fig The growth inhibition of BCR-ABL negative patient cells evaluated by MTT assay (A-E) and trypan blue exclusion assay (F-J). Cells were transfected with 1.2PEI-Lau8 polymer/CTRL and STAT5A siRNA complexes at ratio of 6:1 and 30 and 60 nM siRNA concentrations for 3 days. In MTT assay, cell growth inhibition was expressed relative to non-treated cells (taken as 100%). Furthermore, live cells were counted by trypan blue exclusion assay. Y-axis shows the live cell count (x 10^5) per ml. The data are the mean ± SD. (n = 3) *: p<0.05, **: p<0.01, ***: p<0.001 compared with the complexes with Control siRNA. (K). Summary of significant differences in MTT and cell counts in ALL primary cells.
(PDF)

S1 File (XLSX)
