# Supplementary figures and images for: Therapeutic delivery of siRNA with polymeric carriers to down-regulate STAT5A expression in high-risk B-cell acute lymphoblastic leukemia (B-ALL)
Source: PLoS One. 2021 Jun 22;16(6):e0251719. doi: 10.1371/journal.pone.0251719 (PMC8219370; doi:10.1371/journal.pone.0251719)

SUPPORTING INFORMATION

SUPPLEMENTARY FIGURES

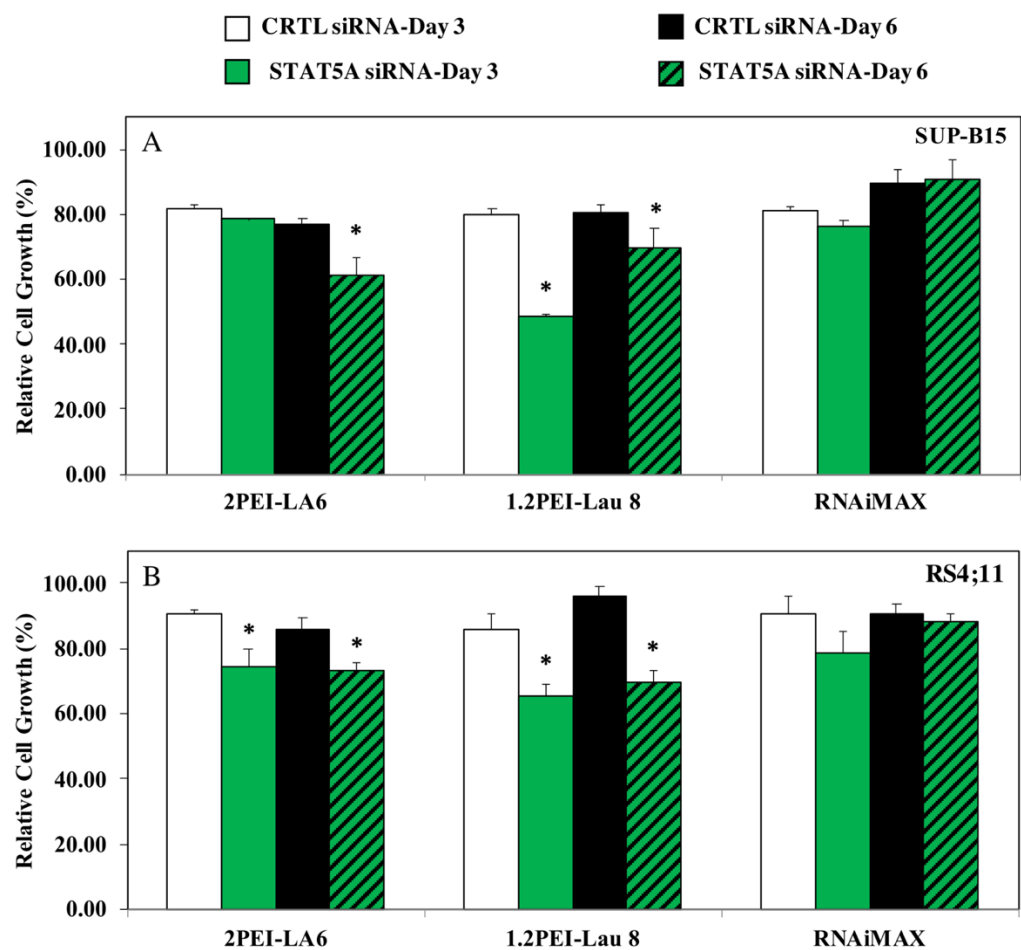

S1 Fig.

Supplement: S1 Fig — Effect of siRNA/polymer complexes on proliferation of SUP-B15 (A) and RS4;11 (B) cells. Cells were treated with polymer/siRNA ratio of 6:1 and 60 nM of Control/STAT5A siRNA complexes for 3 and 6 days and cell growth inhibition was assessed by the MTT Assay and expressed relative to non-treated cells (taken as 100%). The data are the mean ± SD. (n = 3) *p ≤ 0.01 compared with the complexes with Control siRNA. (PDF) [file pone.0251719.s001.pdf]
