# Supplementary material for: Therapeutic delivery of siRNA with polymeric carriers to down-regulate STAT5A expression in high-risk B-cell acute lymphoblastic leukemia (B-ALL)
Source: PLoS One. 2021 Jun 22;16(6):e0251719. doi: 10.1371/journal.pone.0251719 (PMC8219370; doi:10.1371/journal.pone.0251719)
Supplement: S2 Fig — Effects of treatment with STAT5A and BCR-ABL siRNA/polymer complexes on STAT5A (A and B) and BCR-ABL (C and D) mRNA expression in ALL primary cells. mRNA levels were assessed 3 days after transfection at siRNA concentrations of 30 and 60 nM and 1.2PEI-Lau8 polymer:siRNA ratio of 6 by qPCR. (n = 3) (E) Summary of statistical analysis for the results from qPCR results of combination treatment. (PDF) [file pone.0251719.s002.pdf]

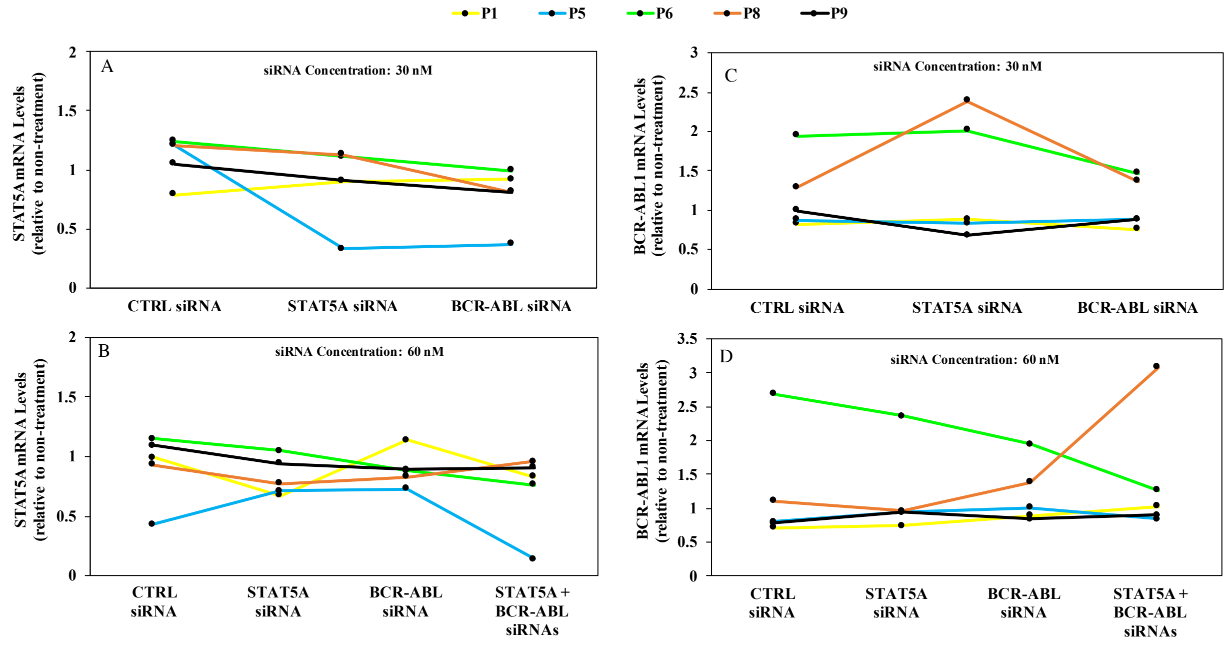

E

	qPCR with STAT5A and BCR-ABL siRNA combination										
Time point	3 Days										
siRNA type and concentration	STAT5A 30 nM		BCR-ABL 30 nM		STAT5A 60 nM		BCR-ABL 60 nM		STAT5A + 30 nM +	BCR-ABL 30 nM	Patient subtype/ Genetic Abnormality
Polymer Group	1.2PEI-Lau8										
mRNA	STAT5A	BCR-ABL	STAT5A	BCR-ABL	STAT5A	BCR-ABL	STAT5A	BCR-ABL	STAT5A	BCR-ABL	
P1	ns	ns	ns	ns	++	ns	ns	ns	+++	ns	BCR-ABL1 Positive (b2a2, P210)
P5	++	ns	++	ns	ns	ns	ns	ns	+	ns	BCR-ABL1 Positive (e1a2, P190)
P6	ns	ns	ns	ns	ns	ns	ns	ns	ns	ns	BCR-ABL1 Positive (b3a2, P210)
P8	ns	ns	ns	ns	n/a	ns	ns	ns	ns	ns	BCR-ABL1 Positive (b3a2, P210)
P9	ns	ns	ns	ns	n/a	ns	+	ns	ns	ns	BCR-ABL1 Positive (e1a2, P190)

ns: not significant, n/a: not available, +: p<0.05 (one-side t-test), ++: p<0.01 (one-side t-test), +++: p<0.001 (one-side t-test)

S2 Fig.
