# Supplementary material for: Therapeutic delivery of siRNA with polymeric carriers to down-regulate STAT5A expression in high-risk B-cell acute lymphoblastic leukemia (B-ALL)
Source: PLoS One. 2021 Jun 22;16(6):e0251719. doi: 10.1371/journal.pone.0251719 (PMC8219370; doi:10.1371/journal.pone.0251719)
Supplement: S3 Fig — The growth inhibition of BCR-ABL negative patient cells evaluated by MTT assay (A-E) and trypan blue exclusion assay (F-J). Cells were transfected with 1.2PEI-Lau8 polymer/CTRL and STAT5A siRNA complexes at ratio of 6:1 and 30 and 60 nM siRNA concentrations for 3 days. In MTT assay, cell growth inhibition was expressed relative to non-treated cells (taken as 100%). Furthermore, live cells were counted by trypan blue exclusion assay. Y-axis shows the live cell count (x 10^5) per ml. The data are the mean ± SD. (n = 3) *: p<0.05, **: p<0.01, ***: p<0.001 compared with the complexes with Control siRNA. (K). Summary of significant differences in MTT and cell counts in ALL primary cells. (PDF) [file pone.0251719.s003.pdf]

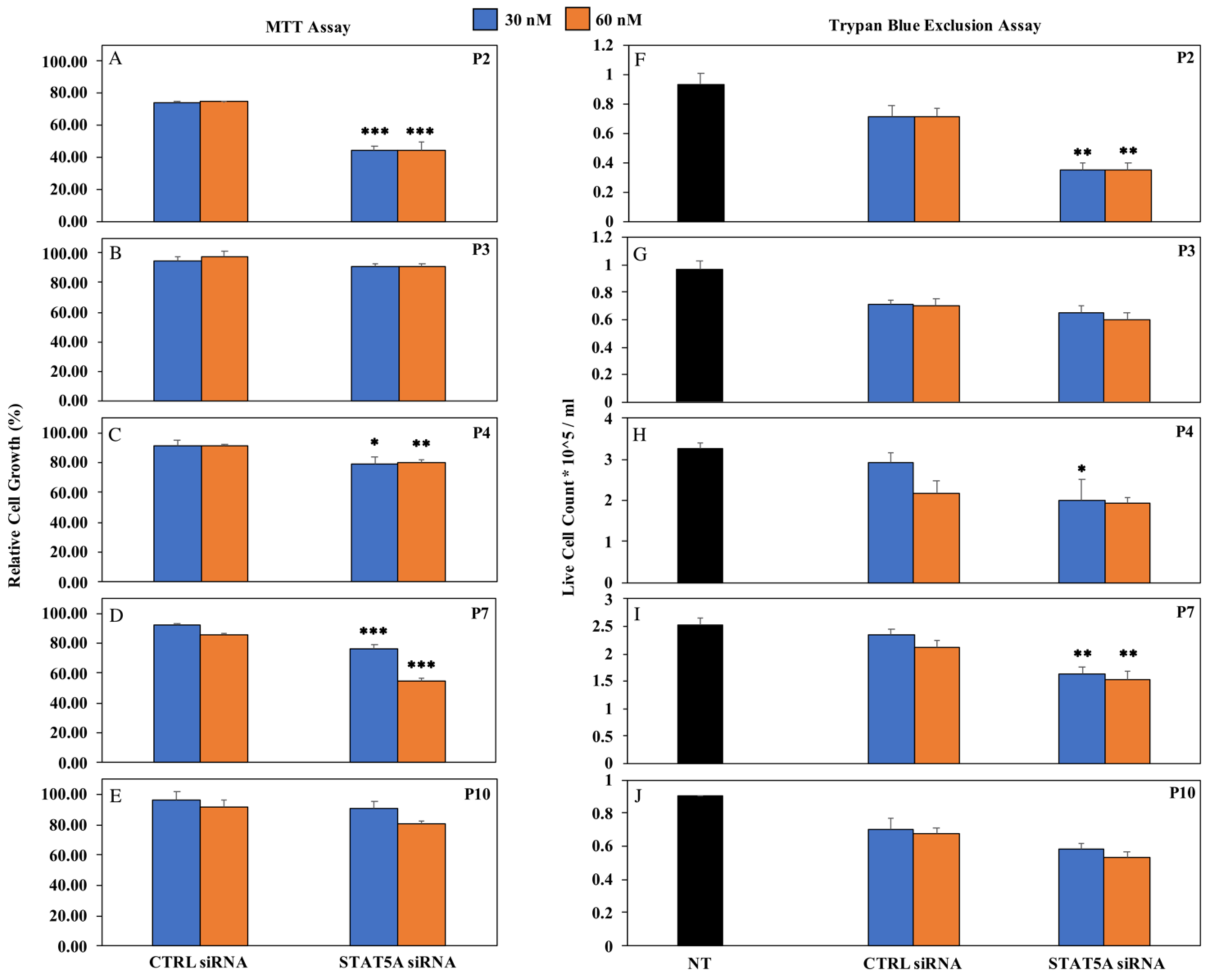

K	MTT		Cell Counts		
Time point	3 Days		3 Days		
siRNA type and concentration	STAT5A 30 nM	STAT5A 60 nM	STAT5A 30 nM	STAT5A 60 nM	Patient subtype/ Genetic Abnormality
Polymer Group	1.2PEI-Lau8		1.2PEI-Lau8		
P2	+ +	+++	+ +	+ +	BCR-ABL1 Negative
P3	ns	ns	ns	ns	BCR-ABL1 Negative, ABL mutated
P4	+	+ +	+	ns	MLL-AF4 t(4;11), MLL rearranged
P7	+++	+++	+ +	+ +	t(1;19)
P10	ns	ns	ns	ns	Cytogenetics failed

S3 Fig.
